# Recent Advances and Future Directions in Thermal, Electrical, and Mechanical Properties of Polymer Composites

**DOI:** 10.3390/polym17172419

**Published:** 2025-09-06

**Authors:** Gabriel Pinto, Victoria Alcázar, Marina P. Arrieta

**Affiliations:** 1 Departamento de Ingeniería Química Industrial y del Medio Ambiente, Escuela Técnica Superior de Ingenieros Industriales, Universidad Politécnica de Madrid (ETSII-UPM), José Gutiérrez Abascal 2, 28006 Madrid, Spain; mariavictoria.alcazar@upm.es (V.A.); m.arrieta@upm.es (M.P.A.); 2 Grupo de Investigación: Polímeros, Caracterización y Aplicaciones (POLCA), 28006 Madrid, Spain

Polymer composites continue to redefine the boundaries of what materials can achieve. Their inherent versatility, particularly in tailoring thermal, electrical, and mechanical properties, makes them indispensable across key technological sectors—from energy storage to electronics, biomedicine, packaging, and construction [1,2]. The modern demand for lightweight, multifunctional, and sustainable materials has accelerated innovation in this field, bridging fundamental research with real-world applications.

Recent decades have witnessed tremendous growth in the development of composites and nanocomposites that combine polymer matrices with a wide range of fillers, including carbon-based nanomaterials, natural fibers, metal oxides, and hybrid structures [3,4,5]. A key challenge has been to achieve property enhancement through optimal filler dispersion, orientation, and interfacial compatibility, without sacrificing processability, cost-effectiveness, or environmental sustainability. In electrically insulating polymers, for instance, the transition to conductive behavior often relies on percolation thresholds, a phenomenon that remains under active investigation [6,7]. Foundational works, such as the review on critical filler concentrations for electroconductive polymer composites [8], remain essential to frame the progress achieved during the last decades. For thermal applications, predicting degradation pathways and long-term stability is crucial—particularly in composites designed for energy or outdoor use [9].

This Special Issue, Advances in Thermal, Electrical and Mechanical Properties of Polymer Composites, offers a representative and forward-looking collection of 16 peer-reviewed articles. These contributions encompass fundamental insights, modeling strategies, novel material systems, and application-oriented studies, highlighting how the design and processing of composites can be optimized to deliver multifunctionality and performance.

Several contributions address sustainable and biodegradable systems. Fartash Naeimi et al. [10] developed hemp-reinforced starch/agar composites and used finite element simulations together with machine learning to predict mechanical performance, showing the potential of combining experimental and computational tools. Rubiano-Navarrete et al. [11] evaluated the effects of UV radiation on polymer composites for beehives, an application directly linked to ecological balance and biodiversity. Duan et al. [12] investigated water vapor and oxygen barrier properties of food packaging paper, achieving impressive reductions in permeability without the need of additional coating layer of a synthetic non-biobased polymeric surface film. Maldonado et al. [13] explored ethylene-scavenging films based on PLA and nano-TiO_2_, offering promising strategies for extending fruit shelf life. Faba et al. [14] used supercritical CO_2_ to foam 3D-printed PLA/CaCO_3_ parts, developing sustainable food-contact foamed materials with enough mechanical and thermal stability for food contact applications.

Electrically conductive composites remain a vibrant research area. Demski et al. [15] enhanced the mechanical and electrical properties of acrylic resins by incorporating chemically modified multi-walled carbon nanotubes (MWCNT), achieving significant conductivity improvements. Mi et al. [16] demonstrated how filler orientation and dispersion govern the anisotropic properties of CNT/polypropylene composites, linking processing conditions with percolation behavior. Tariq et al. [17] applied statistical modeling to optimize filler composition in conductive polypropylene composites for bipolar plates, underscoring the role of design of experiments in materials engineering. Shchegolkov et al. [18] reviewed the potential of CNT-based nanocomposites for strain sensors, self-regulation heating systems, and antistatic coatings, revealing multifunctionality as a major research direction.

In the area of structural and thermal behavior, Yuan et al. [19] analyzed aging in oil-immersed transformer insulation under combined electrical–thermal–mechanical conditions, emphasizing the accelerated deterioration produced by mechanical stress. Fang et al. [20] developed a multi-scale model for the aging performance of particle-filled polymer composites based on matrix of hydroxyl-terminated polybutadiene/toluene diisocyanate and ammonium perchlorate particles (with a diameter of 50–200 μm), establishing relationships between microscopic crosslinking density and macroscopic modulus. Podlozhnyuk et al. [21] synthesized few-layer graphene through high-temperature methods and integrated it into epoxy resins, obtaining substantial improvements in compressive, flexural, and tribological properties. Bouharras et al. [22] developed core-double-shell-structured nanocomposite films based on polyvinylidene fluoride (PVDF). For that, PVDF-grafted BaTiO_3_ nanofillers were incorporated into a PVDF-co-hexafluoropropylene (HFP), enhancing dielectric properties and thermal stability for energy storage devices.

Additive manufacturing emerges as another important theme. Garcia et al. [23] examined graphene oxide nanocomposites for Stereolithography (SLA) 3D printing, conducting extensive mechanical testing under diverse loading modes. Sanchaniya et al. [24] proposed a strategy to improve the transverse mechanical properties of oriented polyacrylonitrile (PAN) nanofiber electrospun mats doping with polyvinyl alcohol (PVA) in different concentrations via using a dip-coating method, confirmed both experimentally and through finite element modeling. These studies show how combining processing innovations with advanced characterization is essential to tailor multifunctionality in next-generation composites.

Complementary works demonstrate the versatility of polymer composites in civil and environmental engineering. Li et al. [25] reported that bentonite-modified acrylate grouting materials significantly improve resistance against washout in high-flow groundwater environments. Such practical applications highlight how polymer composites are not only enabling cutting-edge technologies but also solving urgent infrastructure challenges.

The studies included in this Special Issue collectively show the diversity and maturity of the field. From biodegradable packaging to dielectric films and conductive structures for energy systems, they demonstrate that the properties of polymer composites and nanocomposites can be tuned almost without limit when composition, morphology, dispersion, and processing are optimized. Importantly, predictive approaches—including finite element modeling, response surface methodology, and machine learning—are becoming standard tools in the design of polymer composites, reducing trial-and-error and opening pathways to accelerated discovery [26,27].

Looking forward, several key directions can be identified. First, greater integration of multiscale models, from molecular dynamics to continuum mechanics, will enhance predictive accuracy for composite performance [28]. Second, the pursuit of sustainable and green composites—those that combine biodegradability with advanced thermal, mechanical, or electrical properties—will likely intensify, especially for electronic, medical, and food packaging applications. Third, recycling and upcycling strategies must be developed in parallel with new composites to align with circular economy principles [29]. Finally, addressing challenges related to large-scale processing, reproducibility, and long-term stability will be critical to transition from laboratory prototypes to industrial adoption.

In conclusion, this Special Issue demonstrates not only the remarkable progress made in polymer composite research but also the emerging opportunities and challenges that define the path forward. It provides a foundation for developing materials that are multifunctional, sustainable, and technologically transformative.
